# Three-Dimensional Shapes and Cell Deformability of Rat Red Blood Cells during and after Asphyxial Cardiac Arrest

**DOI:** 10.1155/2019/6027236

**Published:** 2019-10-15

**Authors:** Hui Jai Lee, SangYun Lee, HyunJoo Park, YongKeun Park, Jonghwan Shin

**Affiliations:** ^1^Department of Emergency Medicine, Seoul Metropolitan Government-Seoul National University Boramae Medical Center, Seoul 07061, Republic of Korea; ^2^Department of Physics, Korea Advanced Institute of Science and Technology, Daejeon 24051, Republic of Korea; ^3^Department of Emergency Medicine, Seoul National University College of Medicine, 103 Daehak-ro, Jongno-gu, Seoul 03080, Republic of Korea

## Abstract

Changes in microcirculation are believed to perform an important role after cardiac arrest. In particular, rheological changes in red blood cells (RBCs) have been observed during and after ischemic-reperfusion injury. Employing three-dimensional laser interferometric microscopy, we investigated three-dimensional shapes and deformability of RBCs during and after asphyxial cardiac arrest in rats at the individual cell level. Rat cardiac arrest was induced by asphyxia. Five rats were maintained for 7 min of no-flow time, and then, cardiopulmonary resuscitation (CPR) was started. Blood samples were obtained before cardiac arrest, during CPR, and 60 min after return of spontaneous circulation (ROSC). Quantitative phase imaging (QPI) techniques based on laser interferometry were used to measure the three-dimensional refractive index (RI) tomograms of the RBC, from which structural and biochemical properties were retrieved. Dynamic membrane fluctuations in the cell membrane were also quantitatively and sensitively measured in order to investigate cell deformability. Mean corpuscular hemoglobin, mean cell volume, mean corpuscular hemoglobin concentration, and red blood cell distribution width remained unchanged during CPR and after ROSC compared with those before cardiac arrest. QPI results revealed that RBC membrane fluctuations, sphericity, and surface area did not change significantly during CPR or after ROSC compared with initial values. In conclusion, no three-dimensional shapes and cell deformability changes in RBCs were detected.

## 1. Introduction

Sudden cardiac arrest is one of the main causes of death worldwide. For decades, various research efforts have improved the survival of OHCA victims. However, the long-term neurologic prognosis of cardiac arrest victims still remains poor [1, 2].

Global I/R injury has important roles in a neurological injury of cardiac arrest victims. Cardiac arrest causes deprivation of blood supply to critical organs such as a brain, kidney, and myocardium. Energy stores in the brain are depleted entirely within the first few minutes of the acute ischemic period after cardiac arrest. Resuscitation and ROSC are essential for preventing the progression of irreversible tissue injuries. However, reperfusion of ischemic tissues results in both a local and a systemic inflammatory response and results in widespread microvascular dysfunction. These I/R injury and subsequent inflammatory responses are called postcardiac arrest syndrome. Although optimizing postcardiac arrest care is emphasized and considered as the missing link of resuscitation, the exact mechanism of postcardiac arrest syndrome is not yet fully established [1–5].

Several recent studies have reported an essential role of RBCs in the modulation of the microcirculatory dysfunctions [6–13]. Previous experimental and clinical studies showed changes in morphology and rheological behavior of RBCs in various clinical conditions of I/R injury in sepsis, severe trauma, and critically ill patients [6–16]. These changes contribute the microcirculatory dysfunction and the progress of the tissue injury [11, 17]. Microcirculatory dysfunction is also reported, and impaired microcirculation is related to poor prognosis in postcardiac arrest patients [18, 19].

The exact pathophysiology of microcirculation dysfunction of I-R injury is uncertain. Rheological changes in RBC are regarded as one of the critical factors of microcirculatory failure [7, 11–13, 17]. Cardiac arrest survivors also suffer from global IR injury, and microcirculatory changes also have important roles in the pathologic process of cardiac arrest survivors. Morphology and behavior changes of the RBCs can be present and expected to affect the progress of postcardiac arrest syndrome. However, few things are known about RBCs of postcardiac arrest, and no study has investigated changes in RBC rheology.

Here, we conducted experiments to determine whether rheological changes occur in rat RBCs during and after asphyxial cardiac arrest. In order to precisely and rapidly investigate individual cells, we employed a 3D quantitative phase imaging (QPI) technique and performed the measurements of the 3D shapes and cell deformability of individual RBCs during and after cardiac arrest. Based on the principle of laser interferometry, 3D refractive index (RI) tomograms, cytoplasmic Hb concentration, and dynamic membrane fluctuations of individual RBCs were retrieved. In particular, rheological properties of RBCs were also addressed by analyzing dynamic membrane fluctuations in a cell membrane. With this experiment, we wanted to evaluate the membrane properties, structures, and functional status of RBCs after cardiac arrest.

## 2. Materials and Methods

### 2.1. Animal Preparation

All animal studies were approved by the Institutional Animal Care and Use Committee of SMG-SNU Boramae Medical Center (IACUC no. 2014-0012) and the internal review board of KAIST (IRB Project no. 2012-0128). Five male Sprague Dawley rats (weight, 350–400 g) purchased at Koatech (Pyeongtaek, Korea) were used for experiments. The animals were housed under controlled laboratory conditions (22 ± 2°C; relative humidity, 55–60%), with free access to food and water before the experiment. The rats underwent an acclimatization period of 14 days before using in experiments.

### 2.2. Induction of Cardiac Arrest and Resuscitation Procedures

The animals were anesthetized with an intramuscular injection of zolazepam and tiletamine (Zoletil; Virbac AH, Fort Worth, TX, USA) and maintained on isoflurane (Choongwae Pharm, Seoul, Korea). Endotracheal intubation was done using a 16-gauge catheter (BD Instyle™ Autoguard™; Becton-Dickinson, Parsippany, NJ, USA) and assisted with a mechanical ventilator (tidal volume, 2 mL; respiratory rate, 60/min; FiO_2_, 0.21; Harvard rodent ventilator model 557058, Harvard Apparatus, Holliston, MA, USA). The right femoral artery was cannulated to monitor blood pressure continuously and to sample blood. A tail vein was cannulated for drug administration. The ECG was recorded with the aid of subcutaneous patches. The ECG and femoral arterial blood pressure were recorded continuously on a monitor (DASH 4000, General Electric HealthCare Products, Arlington Heights, IL, USA) for subsequent analyses. Core temperature was measured with a rectal probe and controlled at 36.5 ± 1.0°C using a warmer. Then, isoflurane inhalation was discontinued, and the animals were stabilized for 10 min.

**Figure 1:**
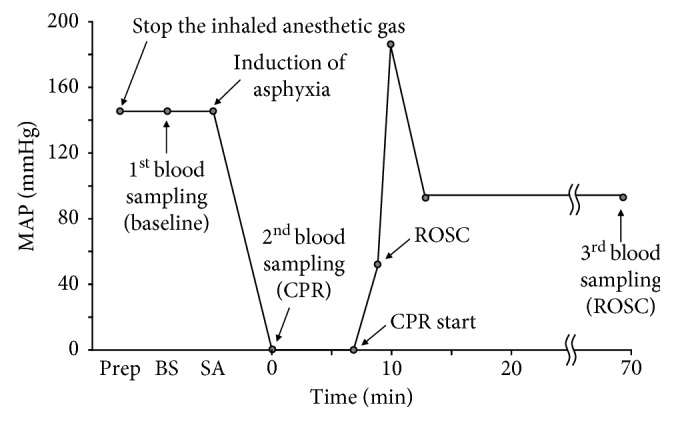
The change of mean arterial pressure in the asphyxial cardiac arrest model and time of blood sampling. MAP: mean arterial pressure; Prep: preparation; BA: baseline sampling; SA: start of asphyxia; ROSC: return of spontaneous circulation.

As depicted in Figure1, asphyxia was induced by suspending ventilation for 10 min, and measurement of baseline parameters was begun by administering vecuronium (Reyon Pharm, Seoul, Republic of Korea) (0.05 mg/kg), stopping the ventilator, and kinking the ventilator connecting tube. Cardiac arrest was defined as MAP <20 mmHg and was maintained for 7 min. Then, CPR was started. The animals received intravenous epinephrine (0.005 mg/kg) prior to CPR. CPR included mechanical ventilation with 100% O_2_ and 200 external manual chest compressions/min. ROSC was defined as a pulsatile MAP >50 mmHg. After ROSC, postresuscitation care was performed by administering sodium bicarbonate (1.0 mEq/kg) and changing the mechanical ventilator setting to achieve a respiratory rate of 60–70/min and to maintain the normocapnia according to our previous pilot study. Temperature control was stopped during CPR and restarted after ROSC.

### 2.3. Blood Sampling and Analyses

Three blood samples were obtained before cardiac arrest, during CPR (at 30 sec after initiation of CPR), and 60 min after ROSC (Figure 1). A total of 1.5 ml of blood was obtained each time and divided into one heparin-coated syringe and two tubes. The syringe (0.5 mL) was used to determine blood gases immediately after blood sampling using RAPIDPoint 405 (Siemens Healthcare, Erlangen, Germany), the one tube (0.5 mL) was used to check the CBC, and the other was used to check RBC rheology. The same volume of saline was infused after each blood sample was obtained. The first tube was sent to an external laboratory (Green Cross Laboratories, Gyeonggi-do, Korea) for CBC. The second tube was sent to the KAIST for the RBC rheological analysis.

### 2.4. Measurements and Outcome

We recorded the critical times during the experiment in the asphyxial cardiac arrest rat model, including the induction time of cardiac arrest and CPR time. All the blood samples were analyzed for CBC and blood gases, including MCV, MCH, MCHC, RDW, pH, PCO_2_, PO_2_, and HCO_3_. Rat RBCs were collected in vacutainer tubes containing ethylenediaminetetraacetic acid to prevent blood clotting and quickly transported safely to the KAIST. We sent another blood sample with the study samples to determine any changes in the RBCs due to transport. The rat RBCs were centrifuged immediately upon arrival at KAIST at 2000 ×*g* at 10°C for 10 min to separate the RBCs from the plasma. The RBCs were washed three times with PBS.

All blood samples sent to KAIST were analyzed for CHC, CM, CV, membrane fluctuations, surface area, and sphericity using cDOT. All the parameters are measured within 10 hours after collections of samples [20].

### 2.5. Common-Path Diffraction Optical Tomography (cDOT)

We used common-path diffraction optical tomography (cDOT) to quantitatively and noninvasively measure the undulatory dynamics of the RBC membranes [21, 22]. Based on the principle of common-path laser interferometry and optical diffraction tomography, cDOT reconstructs the 3D RI tomogram of individual cells without labeling or other preparation processes. This label-free imaging capability makes the present method well suited for the application in emergency medicine. The 3D RI tomogram of a sample is reconstructed from multiple 2D phase distribution of the sample obtained with various illumination angles. This reconstruction process is done by solving an inverse problem of light propagations, also known as Helmholtz differential equation [23, 24]. This principle of optical diffraction tomography is an optical analog to X-ray computed tomography (CT) [25]. X-ray CT reconstructs the 3D absorptivity map of a human body, from multiple 2D projection images obtained with various illumination angles. In contrast, optical diffraction tomography reconstructs the 3D (RI) map of a cell, from multiple 2D projection images with various illumination angles. Because cDOT realizes the principle of optical diffraction tomography in common-path laser interferometry, cDOT provides the capability of highly sensitive measurements for 3D RI tomography [26, 27]. cDOT has shown potentials for the study of RBCs, including correlative analysis [21] and babesiosis [28].

A diode-pumped solid-state laser (*λ* = 532 nm, 50 mW, Cobolt Co., Solna, Sweden) was used as an illumination source for an inverted microscope. The microscope was equipped with a 60x oil immersion objective lens (1.42 NA) that facilitates diffraction-limited transverse resolution of about 200 nm. The overall magnification of the system was approximately 250x with the additional relay optics used outside the microscope. An sCMOS camera (Neo sCMOS, ANDOR Inc., Northern Ireland, U.K.) was used to image the interferograms. Employing common‐path laser interferometry, cDOT provides highly stable full-field quantitative phase images of RBCs. The optical path-length stability was 2.4 mrad, which corresponds to membrane displacement of 3.3 nm. The details on the optical instrumentation and 3D reconstruction algorithms can be found elsewhere [29–31].

### 2.6. Analyses of the Red Cell Indices

Morphological (volume, surface area, and sphericity), biochemical (Hb concentration and content), and mechanical (dynamic membrane fluctuation) properties were retrieved from the measured 3D RI maps and 2D dynamic optical phase delay images.

To measure the morphological parameters, we used the 3D RI maps of individual RBCs measured using cDOT, *n*(*x*, *y*, *z*). The cell volume was calculated by integrating all voxels inside individual RBCs. The size of each voxel (approx. 50 nm × 50 nm × 50 nm) was significantly smaller than the diffraction-limited size. The volume corresponding to the RBC cytoplasm was selected by RI higher than the threshold (*n*_thresh_ = 1.363). The total number of voxels was then counted to obtain the cell volume, considering the lateral magnification of the optical system. Next, the surface area of the RBC membrane was retrieved from the isosurfaces of individual RBCs. The surface area of the isosurface is measured through the summation of the areas of all the patch faces, which are broken down into small triangular pieces. In addition, the sphericity SI, a dimensionless quantity ranging from 0 to 1, was obtained by calculating SI=*π*^1/3^(6*V*)^2/3^/*A*, where the *V* is the volume and *A* is the surface area. A sphericity of 1 is close to a perfect sphere, and 0 is flat.

The Hb concentration of individual RBCs was calculated from the measured 3D RI maps of RBC cytoplasm. The Hb concentration is linearly proportional to the RI contrast, Δ*n*, between an RBC and a surrounding medium as, [Hb]=*α* · Δ*n*, where *α* is a refraction increment for Hb (0.2 mL/g) [32–34]. The Hb content in individual RBCs was then obtained by multiplying the cell volume by the Hb concentration.

From a set of the measured 2D optical phase delay images of each RBC Δ*ϕ*(*x*, *y*, *t*) by cDOT, the dynamic cell height maps *h*(*x*, *y*; *t*) were retrieved using the RI contrast Δ*n*=〈*n*_RBC_〉 − *n*_medium_ between the RBC cytoplasm 〈*n*_RBC_〉 and surrounding medium *n*_medium_, as *h*(*x*, *y*; *t*)=[*λ*/(2*π* · 〈Δ*n*〉)] · Δ*ϕ*(*x*, *y*; *t*). Then, the representative 2D height profile of the corresponding RBC 〈*h*(*x*, *y*; *t*)〉_*t*_ was obtained by taking a time average of the dynamic cell height maps. To quantitatively address the mechanical deformability of the membrane of individual RBCs during CPR and ROSC, the RMS of cell height displacement was calculated at each point on the cell and then averaged over the cell area, as Δ*h*=〈[*h*(*x*, *y*; *t*) − 〈*h*(*x*, *y*)〉^2^]〉^1/2^, where 〈·〉 denotes a spatiotemporal average.

### 2.7. Statistical Analysis

The normality of the data distributions was assessed with the Kolmogorov–Smirnov test. Normally distributed, continuous data are expressed as mean ± standard deviation. Nonnormally distributed data are expressed as median (interquartile ranges). Student's *t*-test was used to compare normally distributed data. A *P* value <0.05 was considered statistically significant. The Kruskal–Wallis test with the Mann–Whitney *U* post hoc test and Bonferroni correction were used for nonnormally distributed data among three groups, and a *P* value <0.017 was considered statistically significant. All analyses were performed using IBM SPSS 20 (IBM Corp., Armonk, NY, USA).

## 3. Results

### 3.1. Comparison of the Value of CBC and Arterial Blood Gas after Cardiac Arrest

To investigate the alterations in RBCs, we measured CBC values for rats undergo cardiac arrest induced by asphyxia. All rats were maintained for 7 min of no-flow time after mean arterial pressure was <20 mmHg, and then CPR was started. We performed postcardiac arrest care of rats after ROSC. Blood samples were obtained before cardiac arrest, during CPR, and 60 min after ROSC to check arterial blood gases and CBC.

Our result shows that the CBC values, including MCV, MCH, and MCHC, were not statistically different before cardiac arrest, during CPR, and 60 min after ROSC (Table 1). pH decreased during CPR (7.05 (7.00–7.11)) and was restored after ROSC (7.21 (7.12–7.28)) compared with that at baseline (7.32 (7.19–7.36)). PCO_2_ increased during CPR (68.9 (59.8–77.2) mmHg) compared with that at baseline (39.0 (33.5–42.2) mmHg). PO_2_ decreased during CPR (52 [35–47] mmHg)and was restored after ROSC (155 (96-170) mmHg). HCO_3_ did not change significantly (*P* value = 0.859). The mean induction time to cardiac arrest was 2.0 ± 0.5 min, and the mean CPR duration was 2.0 ± 1.0 min. Mean rat rectal temperature before the cardiac arrest was 36.4 ± 0.3°C and was 36.5 ± 1.1°C after ROSC.

### 3.2. Morphological and Biochemical Alterations in Individual RBCs

**Figure 2:**
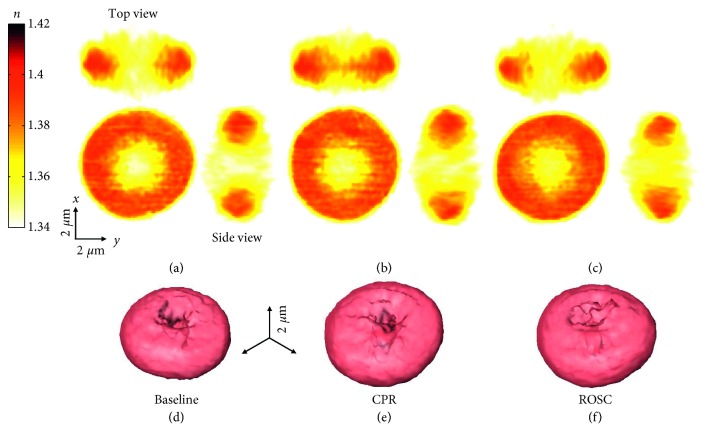
The reconstructed 3D RI distribution of RBCs. (a–c) Three 2D cross sections (*x*-*y*, *x*-*z*, and *y*-*z* planes) of the 3D RI map for the representative RBC from baseline, CPR, and ROSC groups, respectively. (d–f) 3D rendered RI isosurfaces of the corresponding three representative RBCs.

**Figure 3:**
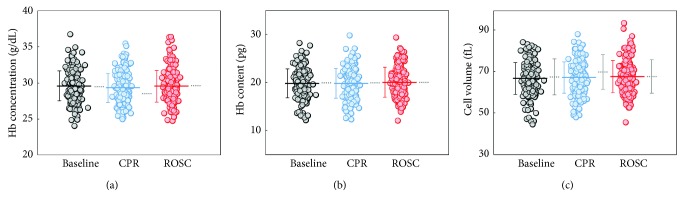
Red cell parameters of individual RBCs. (a) Hb concentration, (b) Hb content, and (c) cell volume of individual RBCs in groups of the baseline, CPR, and ROSC, respectively. *∗* indicates *P* value <0.05. Each symbol represents individual RBC measurements, and the horizontal lines are mean values with vertical lines of standard deviation error bars. Horizontal gray dotted lines in (a)–(c) correspond to mean cellular Hb concentration (MCHC), mean cellular Hb content (MCH), and mean cellular volume (MCV), respectively.

To investigate structural alterations after CPR and ROSC at the individual cell levels, we measured 3D RI tomograms of RBCs, *n*(*x*, *y*, *z*), using cDOT. For visualization purpose, the three cross-sectional slices (*x-y*, *x-z*, and *y-z* planes) of the reconstructed RI tomogram and the corresponding RI isosurface of the representative RBC in each RBC group are shown in Figure 2. In detail, for example, the 2D *x*-*y* cross-sectional slice of the RBC corresponds to *n*(*x*, *y*, *z*=*z*_0_), where *z*_0_ is a manually determined *z*-coordinate of the RBC center plane. Then, from the 3D RI tomogram of individual RBCs, important red cell indices including Hb concentration, Hb content, and cell volume are retrieved, as shown in Figure 3 (for more detailed information, see *Materials and Methods*). The number of quantitatively measured and analyzed RBC in accordance with the sample time was 197 before cardiac arrest, 196 during CPR, and 197 after ROSC.

The Hb concentrations of RBCs in each group are shown in Figure 3(a). Hb concentration of individual RBCs was obtained from the tomographic measurements of RI values of RBC cytoplasm because RI contrast, Δ*n*, between an RBC and a surrounding media is linearly proportional to Hb protein in RBC cytoplasm [21, 28, 48, 58].

Conducted statistical analyses did not guarantee any alteration in Hb concentration of measured RBCs of rats in both CPR and ROSC periods (Figure 3(a)). The mean values of the Hb concentration are 29.6 ± 2.1, 29.3 ± 2.0, and 29.5 ± 2.2 *g*/dL for RBCs in the baseline, CPR, and ROSC groups, respectively. This result is consistent with the CBC test (Table 1).

The Hb content or cellular dry mass of RBCs, i.e., the nonaqueous materials inside RBC, was obtained using the cDOT measurements [34]. The Hb content of RBCs does not exhibit significant changes during CPR and ROSC (Figure 3(b)). The mean values of the Hb content are 19.8 ± 3.0, 19.8 ± 3.1, and 20.0 ± 3.1 pg for RBCs in the baseline, CPR, and ROSC groups, respectively.

In addition, the cell volume does not exhibit statistical changes during CPR and ROSC, neither (Figure 3(c)). The cell volume was obtained by integrating voxels corresponding to cells in the measured 3D RI tomograms. The mean values of the cell volume are 66.7 ± 7.7, 67.2 ± 7.6, and 67.5 ± 7.7 fL for RBCs in the baseline, CPR, and ROSC groups, respectively. This result of the measurements of Hb content and cell volume agrees well with the CBC test (Table 1).

### 3.3. Deformability of Individual RBCs during CPR and ROSC

In order to investigate the rheological changes of individual RBCs during CPR and ROSC, the dynamic fluctuations in RBC membranes were quantitatively measured. Dynamic membrane fluctuations in RBCs, consisting of submicron displacements in the cell membrane, are strongly correlated with deformability and rheological properties of RBC [49, 50]. Previously, dynamic membrane fluctuations in RBC have been investigated for the study of alteration in rheological properties, caused by morphology [51], ATP [52, 53], osmotic pressure [27], malaria infection [54, 55], stored blood [56], cord blood [56], and diabetes mellitus [20].

**Figure 4:**
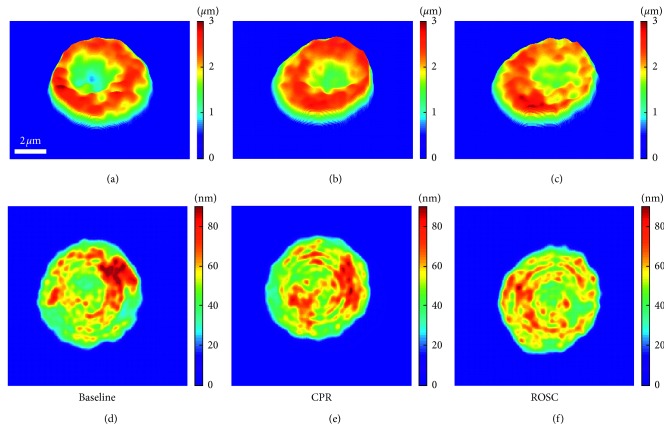
(a)–(c) 2D height profiles of the representative RBC from baseline, CPR, and ROSC groups, respectively. (d)–(f) 2D RMC fluctuation maps of the corresponding three representative RBCs.

The RBC maps in the first row in Figure 4 depict the 2D height profiles of the three representative RBCs in the baseline, CPR, and ROSC groups, respectively. Then, in order to visualize the dynamic fluctuating motion of RBC membranes, the root-mean-square (RMS) height fluctuation maps of corresponding RBCs were obtained from the time series of 2D height profiles, as shown in the second row in Figure 4 (for details, see *Materials and Methods*).

**Figure 5:**
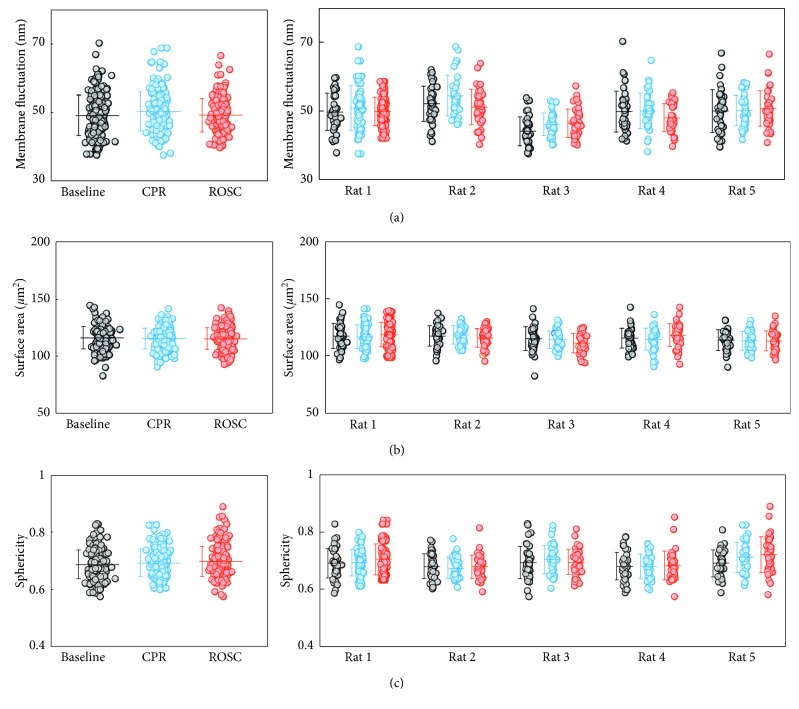
Dynamic membrane fluctuations, sphericity, and surface area of individual RBCs using CPR and ROSC. (a) RMC fluctuation of the cell membrane of individual RBCs, (b) cell surface area, and (c) sphericity. Each symbol represents individual RBC measurements, and the horizontal lines are mean values with vertical lines of standard deviation error bars.

The averaged RMS height fluctuations of individual RBCs during the baseline, CPR, and ROSC groups are shown in Figure 5(a). Experiments were conducted on five rats to address animal-to-animal variations. For each rat, a total of 120 RBCs were measured using cDOT. Although there exist variations between rats, the values of the RMS height fluctuation of RBCs do not exhibit significant alterations during CPR and ROSC, in comparison with those of the baseline group. The mean values of the averaged RMS height fluctuation are 49.1 ± 6.0, 50.3 ± 5.7, and 49.2 ± 4.9 nm for RBCs in the baseline, CPR, and ROSC groups, respectively. This result clearly indicates that the deformability of individual RBCs is not significantly changed during the CPR and ROSC.

To further analyze the alterations in morphologies of individual RBCs, the values of the surface area and sphericity were retrieved. The result is shown in Figure 5(b) and it is clearly seen that there is no statistical difference in the cell surface area between RBCs in the baseline, CPR, and ROSC groups. The mean values of the cell surface area are 115.9 ± 9.6, 115.5 ± 8.9, and 115.3 ± 9.6 *μ*m2 for RBCs in the baseline, CPR, and ROSC groups, respectively. These results exclude the possibility of significant microvesiculation during the CPR and ROSC.

From the measured cell volume and surface area, sphericity, a unit-less parameter indicating the roundness of a cell, was calculated. The result is shown in Figure 5(c). These results show that the overall roundness of individual cells is not significantly altered during CPR and ROSC. The result can also be expected from the observations that both the cell volume and surface area were not changed during CPR and ROSC.

## 4. Discussion

This study is, to our knowledge, the first study evaluating RBC rheology changes during or after cardiac arrest. No morphologic, biochemical, and deformability changes occurred in rat RBCs during and 60 min after asphyxial cardiac arrest compared with those at baseline. The rat RBCs remained unchanged throughout the experimental period. The duration of arrest time including induction is typically <9 min in many experimental preclinical cardiac arrest animal models [35–41, 57]. Brain damage often occurs due to asphyxia after 7 min in the cardiac arrest rat model. Many studies have investigated the effects of various interventions using this model. The total mean asphyxia time with cardiac arrest was about 9 min in our study. No-flow time, which is true arrest time, was 7 min. Thus, 7 min of cardiac arrest was sufficient to cause systemic ischemia.

In one previous study, ischemia has been induced in rat hind limbs by occluding the femoral artery for 10 min, followed by reperfusion. RBC deformability as measured by ektacytometry was significantly impaired immediately after the ischemic period in blood samples obtained from the femoral vein of the ischemic limb, but no significant difference was observed after 15 min of reperfusion [10]. In another study, I/R was produced by clipping the superior mesenteric artery for 30 min, and the area was reperfused for 60 min before extermination. Blood samples were taken from the caudal caval vein and from the portal vein before ischemia, 1 min before and after clip removal, and after 15, 30, and 60 min of reperfusion. RBC deformability was determined by slit-flow ektacytometry based on the erythrocyte laser diffraction image analysis at various levels of shear stress. RBC deformability worsened significantly in the I/R group during the experimental period compared to that at baseline and in the sham group [16].

Some studies have been performed on RBC rheology in critically ill patients or those with sepsis. A previous review article reported that changes in RBC rheology can contribute to microvascular injury and the impaired oxygen supply seen in patients with sepsis [8]. Many factors are involved in RBC rheology, including nitric oxide, reactive oxygen species, altered calcium homeostasis, decreases in ATP reserves, increases in intracellular 2,3 diphosphoglycerate, membrane components (sialic acid), and interactions with white blood cells. These findings suggest that understanding the mechanisms of the changes in RBC rheology in patients with sepsis and their effects on blood flow and oxygen transport may lead to improved patient management and reduced morbidity and mortality. In another study, a total of 196 patients in the ICU (160 without and 36 with sepsis) and 20 healthy volunteers were studied by laser-assisted optical rotational cell analysis within the first 24 hours after ICU admission for an RBC rheological assessment [12]. They showed that early changes in RBC rheology are common in patients in the ICU, particularly in those with sepsis. These changes may contribute to the microcirculatory alterations observed in critically ill patients. Two studies have been performed about changes in RBC rheology over time in critically ill patients with sepsis and their relationships with outcome or prognosis [25, 26]. They concluded that changes in RBC rheology and deformability are useful to estimate the prognosis of patients with sepsis and that reduced RBC deformability over time is associated with a poor outcome.

Early-stage microcirculatory dysfunction is also reported in cardiac arrest victims. Van Genderen et al. reported early microcirculatory dysfunction of postcardiac arrest patients, and it is caused by vasoconstriction due to induced systemic hypothermia [42]. Omar et al. also reported microcirculatory dysfunction in the early stage and there was no statistical relationship between degrees of impairment of microcirculation and levels of cytokines and lactate [19]. Inflammatory responses are regarded as one of the most important factors which induce hemorheological changes of RBC [17]. Properties of RBC were not changed at 60 min after ROSC in our study. Early microcirculatory dysfunction of postcardiac arrest may not relate to inflammatory response and RBC rheology changes.

DPM is a QPI technique and has been widely used to investigate RBC deformability [27–29]. Dynamic membrane fluctuations, consisting of submicron displacement in the cell membrane, are strongly correlated with RBC deformability and rheological properties [30, 31]. Using DPM, several alterations in deformability of RBCs have been investigated, including the effects of ATP and the behavior of RBC deformability in response to various osmotic pressures.

The biomechanical properties of RBCs are crucial for their physiology. This essential deformability is, in turn, affected by various pathophysiological conditions. Temperature plays an important role in deformability of RBCs [43, 54]. Extracellular media of different osmolalities causes changes in RBC morphology and, thus, deformability [44]. The presence of ATP is essential for the RBC to maintain their biconcave shape and significantly affects RBC deformability [45, 51]. Malaria infection results in significant modifications to the rheological properties of host RBCs. Membrane fluctuation measurements show an increased shear modulus and a loss of deformability in malaria-infected RBCs [46, 54]. Quantitative phase microscopy has been used to measure a decrease in membrane fluctuations in sickled RBCs [47]. Diabetic RBCs showed diminished membrane fluctuation and reduced deformability [20].

Several limitations of our study should be mentioned. First, we used a nonhuman, small rodent, rat cardiac arrest model. Some differences may occur between rat RBCs and human RBCs during global ischemia, CPR, and after ROSC. Second, we did not have samples more than 60 minutes after ROSC because of time limitations to conduct the RBC analyses. However, as mentioned above in the limb ischemia model, RBC rheology changes were started earlier than 60 minutes. Further study is needed to analyze blood samples for an extended time period. Third, we did not evaluate RBCs of extended durations of arrest time. Fourth, we did not measure inflammatory cytokines and microvascular flow which can reflect the systemic inflammation and tissue perfusion status. Fifth, other parameters such as blood viscosity, shear rate, and plasma flow rate were not directly evaluated. Also, in vivo analysis of the rheological change was not evaluated.

## 5. Conclusion

Three-dimensional shapes and cell deformability changes of the RBCs were not observed in a 7 min cardiac arrest rat model of asphyxia, such as fluctuations, surface area, or sphericity, during CPR and 60 min after ROSC using cDOT. Additional research is needed to determine whether RBCs undergo rheological changes in patients suffering from cardiac arrest.

## Figures and Tables

**Table 1:** The comparison of the value of the CBC and arterial blood gas analysis according to the sample time.

	Baseline	During CPR	60 min after ROSC	*P* value
pH	7.32 (7.19–7.36)	7.05 (7.00–7.11)	7.21 (7.12–7.28)	0.011
PCO_2_ (mmHg)	39.0 (33.5–42.2)	68.9 (59.8–77.2)	56.3 (40.2–84.7)	0.018
PO_2_ (mmHg)	312 (240–361)	52 [35–47]	155 (96–170)	0.002
HCO_3_ (mEq/L)	17.8 (15.1–20.1)	18.6 (16.6–20.8)	17.8 (16.4–19.4)	0.859
Hemoglobin (g/dL)	14.3 (14.0–14.6)	13.7 (11.5–14.2)	14.1 (13.0–14.7)	0.223
Hematocrit (%)	48 [36–39]	42 [35–37, 48–57]	47 [35–39, 57]	0.658
MCV (fL)	67.5 (67.7–69.4)	70.8 (70.1–71.6)	67.7 (65.5–69.0)	0.164
MCH (pg)	19.9 (19.8–20.0)	20.1 (19.5–20.2)	20.0 (19.9–20.1)	0.883
MCHC (g/dL)	29.6 (28.8–29.7)	28.5 (28.1–28.5)	29.6 (28.8–30.2)	0.183
RDW (%)	12.8 (12.3–15.6)	12.3 (11.4–12.5)	12.1 (11.8–12.2)	0.227
Body temperature (°C)	36.3 (36.3–36.4)	35.3 (34.5–36.0)	36.8 (36.4–37.0)	0.036

Values are expressed as median with interquartile range. CBC: complete blood count; CPR: cardiopulmonary resuscitation; ROSC: return of spontaneous circulation; MCV: mean corpuscular volume; MCH: mean corpuscular hemoglobin; MCHC: mean corpuscular hemoglobin concentration; RDW: red cell distribution width.

## Data Availability

The data used to support the findings of this study are available from the corresponding author upon request.

## References

[1] Nolan J. P., Neumar R. W., Adrie C. (2008). Post-cardiac arrest syndrome: epidemiology, pathophysiology, treatment, and prognostication. A scientific statement from the international liaison committee on resuscitation; the American heart association emergency cardiovascular care committee; the council on cardiovascular surgery and anesthesia; the council on cardiopulmonary, perioperative, and critical care; the council on clinical cardiology; the council on stroke. *Resuscitation*.

[2] Nolan J. P., Soar J. (2010). Postresuscitation care: entering a new era. *Current Opinion in Critical Care*.

[3] Adrie C., Adib-Conquy M., Laurent I. (2002). Successful cardiopulmonary resuscitation after cardiac arrest as a “sepsis-like” syndrome. *Circulation*.

[4] Adrie C., Laurent I., Monchi M., Cariou A., Dhainaou J.-F., Spaulding C. (2004). Postresuscitation disease after cardiac arrest: a sepsis-like syndrome?. *Current Opinion in Critical Care*.

[5] Siesjö B. K., Siesjö P. (1996). Mechanisms of secondary brain injury. *European Journal of Anaesthesiology*.

[6] Hurd T. C., Dasmahapatra K. S., Rush B. F., Machiedo G. W. (1988). Red blood cell deformability in human and experimental sepsis. *Archives of Surgery*.

[7] Powell R. J., Machiedo G. W., Rush B. F. (1993). Decreased red blood cell deformability and impaired oxygen utilization during human sepsis. *The American Journal of Surgery*.

[8] Astiz M. E., DeGent G. E., Lin R. Y., Rackow E. C. (1995). Microvascular function and rheologic changes in hyperdynamic sepsis. *Critical Care Medicine*.

[9] Baskurt O. K., Gelmont D., Meiselman H. J. (1998). Red blood cell deformability in sepsis. *American Journal of Respiratory and Critical Care Medicine*.

[10] Kayar E., Mat F., Meiselman H. J., Baskurt O. K. (2001). Red blood cell rheological alterations in a rat model of ischemia-reperfusion injury. *Biorheology*.

[11] Piagnerelli M., Boudjeltia K. Z., Vanhaeverbeek M., Vincent J.-L. (2003). Red blood cell rheology in sepsis. *Intensive Care Medicine*.

[12] Moutzouri A. G., Skoutelis A. T., Gogos C. A., Missirlis Y. F., Athanassiou G. M. (2007). Red blood cell deformability in patients with sepsis: a marker for prognosis and monitoring of severity. *Clinical Hemorheology and Microcirculation*.

[13] Reggiori G., Occhipinti G., De Gasperi A., Vincent J.-L., Piagnerelli M. (2009). Early alterations of red blood cell rheology in critically ill patients. *Critical Care Medicine*.

[14] Berezina T. L., Zaets S. B., Machiedo G. W. (2004). Alterations of red blood cell shape in patients with severe trauma. *The Journal of Trauma: Injury, Infection, and Critical Care*.

[15] Nemeth N., Soukup J., Menzel M. (2006). Local and systemic hemorheological effects of cerebral hyper- and hypoperfusion in a porcine model. *Clinical Hemorheology and Microcirculation*.

[16] Brath E., Nemeth N., Kiss F. (2010). Changes of local and systemic hemorheological properties in intestinal ischemia-reperfusion injury in the rat model. *Microsurgery*.

[17] Ince C. (2005). The microcirculation is the motor of sepsis. *Critical Care*.

[18] Mongardon N., Dumas F., Ricome S. (2011). Postcardiac arrest syndrome: from immediate resuscitation to long-term outcome. *Annals of Intensive Care*.

[19] Omar Y. G., Massey M., Andersen L. W. (2013). Sublingual microcirculation is impaired in post-cardiac arrest patients. *Resuscitation*.

[20] Lee S., Park H., Kim K., Sohn Y., Jang S., Park Y. (2017). Refractive index tomograms and dynamic membrane fluctuations of red blood cells from patients with diabetes mellitus. *Scientific Reports*.

[21] Kim Y., Shim H., Kim K., Park H., Jang S., Park Y. (2015). Profiling individual human red blood cells using common-path diffraction optical tomography. *Scientific Reports*.

[22] Shin S., Kim Y., Lee K. Common-path diffraction optical tomography with a low-coherence illumination for reducing speckle noise.

[23] Wolf E. (1969). Three-dimensional structure determination of semi-transparent objects from holographic data. *Optics Communications*.

[24] Park Y., Depeursinge C., Popescu G. (2018). Quantitative phase imaging in biomedicine. *Nature Photonics*.

[25] Kim K., Yoon J., Shin S., Lee S., Yang S.-A., Park Y. (2016). Optical diffraction tomography techniques for the study of cell pathophysiology. *Journal of Biomedical Photonics & Engineering*.

[26] Lee K., Kim K., Jung J. (2013). Quantitative phase imaging techniques for the study of cell pathophysiology: from principles to applications. *Sensors*.

[27] Park Y., Best C. A., Kuriabova T. (2011). Measurement of the nonlinear elasticity of red blood cell membranes. *Physical Review E*.

[28] Park H., Hong S.-H., Kim K. (2015). Characterizations of individual mouse red blood cells parasitized by Babesia microti using 3-D holographic microscopy. *Scientific Reports*.

[29] Kim K., Yoon H., Diez-Silva M., Dao M., Dasari R. R., Park Y. (2013). High-resolution three-dimensional imaging of red blood cells parasitized by Plasmodium falciparum and in situ hemozoin crystals using optical diffraction tomography. *Journal of Biomedical Optics*.

[30] Kim Y., Shim H., Kim K. (2014). Common-path diffraction optical tomography for investigation of three-dimensional structures and dynamics of biological cells. *Optics Express*.

[31] Lim J., Lee K., Jin K. H. (2015). Comparative study of iterative reconstruction algorithms for missing cone problems in optical diffraction tomography. *Optics Express*.

[32] Barer R. (1953). Determination of dry mass, thickness, solid and water concentration in living cells. *Nature*.

[33] Davies H. G., Wilkins M. H. F. (1952). Interference microscopy and mass determination. *Nature*.

[34] Popescu G., Park Y., Lue N. (2008). Optical imaging of cell mass and growth dynamics. *American Journal of Physiology-Cell Physiology*.

[35] Jia X., Koenig M. A., Shin H.-C. (2006). Quantitative EEG and neurological recovery with therapeutic hypothermia after asphyxial cardiac arrest in rats. *Brain Research*.

[36] Albertsmeier M., Teschendorf P., Popp E., Galmbacher R., Vogel P., Böttiger B. W. (2007). Evaluation of a tape removal test to assess neurological deficit after cardiac arrest in rats. *Resuscitation*.

[37] Callaway C. W., Ramos R., Logue E. S., Betz A. E., Wheeler M., Repine M. J. (2008). Brain-derived neurotrophic factor does not improve recovery after cardiac arrest in rats. *Neuroscience Letters*.

[38] Keilhoff G., Schweizer H., John R., Langnaese K., Ebmeyer U. (2011). Minocycline neuroprotection in a rat model of asphyxial cardiac arrest is limited. *Resuscitation*.

[39] Lee J. H., Kim K., Jo Y. H. (2013). Effect of valproic acid on survival and neurologic outcomes in an asphyxial cardiac arrest model of rats. *Resuscitation*.

[40] Ebmeyer U., Esser T., Keilhoff G. (2014). Low-dose nitroglycerine improves outcome after cardiac arrest in rats. *Resuscitation*.

[41] Lu J., Qian H.-Y., Liu L.-J. (2014). Mild hypothermia alleviates excessive autophagy and mitophagy in a rat model of asphyxial cardiac arrest. *Neurological Sciences*.

[42] van Genderen M. E., Lima A., Akkerhuis M., Bakker J., van Bommel J. (2012). Persistent peripheral and microcirculatory perfusion alterations after out-of-hospital cardiac arrest are associated with poor survival. *Critical Care Medicine*.

[43] Waugh R., Evans E. A. (1979). Thermoelasticity of red blood cell membrane. *Biophysical Journal*.

[44] Mohandas N., Clark M. R., Jacobs M. S., Shohet S. B. (1980). Analysis of factors regulating erythrocyte deformability. *Journal of Clinical Investigation*.

[45] Tuvia S., Levin S., Bitler A., Korenstein R. (1998). Mechanical fluctuations of the membrane-skeleton are dependent on F-actin ATPase in human erythrocytes. *The Journal of Cell Biology*.

[46] Fedosov D. A., Lei H., Caswell B., Suresh S., Karniadakis G. E. (2011). Multiscale modeling of red blood cell mechanics and blood flow in malaria. *PLoS Computational Biology*.

[47] Shaked N. T., Satterwhite L. L., Telen M. J., Truskey G. A., Wax A. (2011). Quantitative microscopy and nanoscopy of sickle red blood cells performed by wide field digital interferometry. *Journal of Biomedical Optics*.

[48] Jung J.-H., Jang J., Park Y. (2013). Spectro-refractometry of individual microscopic objects using swept-source quantitative phase imaging. *Analytical Chemistry*.

[49] Popescu G., Park Y., Dasari R. R., Badizadegan K., Feld M. S. (2007). Coherence properties of red blood cell membrane motions. *Physical Review E*.

[50] Popescu G., Park Y., Choi W., Dasari R. R., Feld M. S., Badizadegan K. (2008). Imaging red blood cell dynamics by quantitative phase microscopy. *Blood Cells, Molecules, and Diseases*.

[51] Park Y., Best C. A., Badizadegan K. (2010). Measurement of red blood cell mechanics during morphological changes. *Proceedings of the National Academy of Sciences*.

[52] Park Y., Best C. A., Auth T. (2010). Metabolic remodeling of the human red blood cell membrane. *Proceedings of the National Academy of Sciences*.

[53] Ben-Isaac E., Park Y., Popescu G., Brown F. L. H., Gov N. S., Shokef Y. (2011). Effective temperature of red-blood-cell membrane fluctuations. *Physical Review Letters*.

[54] Park Y., Diez-Silva M., Popescu G. (2008). Refractive index maps and membrane dynamics of human red blood cells parasitized by Plasmodium falciparum. *Proceedings of the National Academy of Sciences*.

[55] Chandramohanadas R., Park Y., Lui L. (2011). Biophysics of malarial parasite exit from infected erythrocytes. *PLoS One*.

[56] Park H., Ahn T., Kim K. (2015). Three-dimensional refractive index tomograms and deformability of individual human red blood cells from cord blood of newborn infants and maternal blood. *Journal of Biomedical Optics*.

[57] Xiao F., Safar P., Radovsky A. (1998). Mild protective and resuscitative hypothermia for asphyxial cardiac arrest in rats. *The American Journal of Emergency Medicine*.

[58] Jang Y., Jang J., Park Y. (2012). Dynamic spectroscopic phase microscopy for quantifying hemoglobin concentration and dynamic membrane fluctuation in red blood cells. *Optics Express*.

